# Crystal structure of a nucleoside model for the inter­strand cross-link formed by the reaction of 2′-de­oxy­guanosine and an abasic site in duplex DNA

**DOI:** 10.1107/S205698901600517X

**Published:** 2016-04-05

**Authors:** Michael J. Catalano, Kasi Viswanatharaju Ruddraraju, Charles L. Barnes, Kent S. Gates

**Affiliations:** a 125 Chemistry Bldg, University of Missouri-Columbia, MO 65211, USA

**Keywords:** crystal structure, purine-6(9*H*)-one, 2′-de­oxy­guanosine, de­oxy-d-ribo­furan­ose, glycosidic linkage, nucleobase, hydrogen bonding

## Abstract

The title compound, 9-[(2*R*,4*S*,5*R*)-4-hy­droxy-5-(hy­droxy­meth­yl)tetra­hydro­furan-2-yl]-2-{[(2*R*,4*S*,5*R*)-4-meth­oxy-5-(meth­oxy­meth­yl)tetra­hydro­furan-2-yl]amino}-1*H*-purin-6(9*H*)-one, C_17_H_25_N_5_O_7_, crystallizes with two independent mol­ecules (*A* and *B*) in the asymmetric unit. In the crystal, the guanosine moieties of mol­ecules *A* and *B* are linked by N—H⋯N and O—H⋯N hydrogen-bonding inter­actions, forming ribbons which are stacked to form columns along [100]. These columns are then linked by O—H⋯O hydrogen bonds between the ribose moieties and numerous C—H⋯O inter­actions to complete the three-dimensional structure.

## Abstract 2

Crystallographic analysis of a nucleoside analog of the 2′-de­oxy­guanosine/abasic site cross-link is presented. This structure corroborates an earlier two-dimensional NMR analysis, concluding that the 2-de­oxy­ribose unit attached at the exocyclic *N* ^2^-amino group of the guanine residue exists in the cyclic amino­glycoside form.

## Chemical context   

Recent work has characterized a structurally novel set of inter­strand DNA–DNA cross-links involving reaction of the ubiquitous DNA abasic lesion with a nucleobase on the opposing strand of the double helix (Catalano *et al.*, 2015 ▸; Dutta *et al.*, 2007 ▸; Gamboa Varela & Gates, 2015 ▸; Johnson *et al.*, 2013 ▸; Price *et al.*, 2014 ▸, 2015 ▸; Yang *et al.*, 2015 ▸; Zhang *et al.*, 2015 ▸). Evidence indicates that the covalent attachment is forged between the anomeric carbon of the abasic sugar and the exocyclic amino group of either a guanine, adenine, or *N* ^4^-amino­cytosine residue (Catalano *et al.*, 2015 ▸; Dutta *et al.*, 2007 ▸; Gamboa Varela & Gates, 2015 ▸; Johnson *et al.*, 2013 ▸; Price *et al.*, 2014 ▸, 2015 ▸; Yang *et al.*, 2015 ▸). This type of glycosidic linkage involving the exocyclic amino group of a nucleobase is reminiscent of that found in the natural products anicemycin, spicamycin, and septacidin (Acton *et al.*, 1977 ▸; Igarashi *et al.*, 2005 ▸; Suzuki *et al.*, 2002 ▸). 
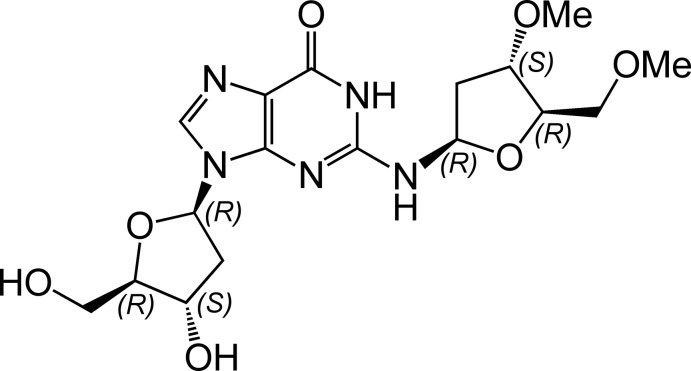


Here we present single crystal X-ray crystallographic analysis of a nucleoside analog, (I), of the 2′-de­oxy­guanosine/abasic site cross-link. This structure corroborates an earlier two-dimensional NMR analysis (Catalano *et al.*, 2015 ▸) concluding that the 2-de­oxy­ribose unit attached at the exocyclic *N* ^2^-amino group of the guanine residue exists in the cyclic amino­glycoside form.

## Structural commentary   

**Figure 1:**
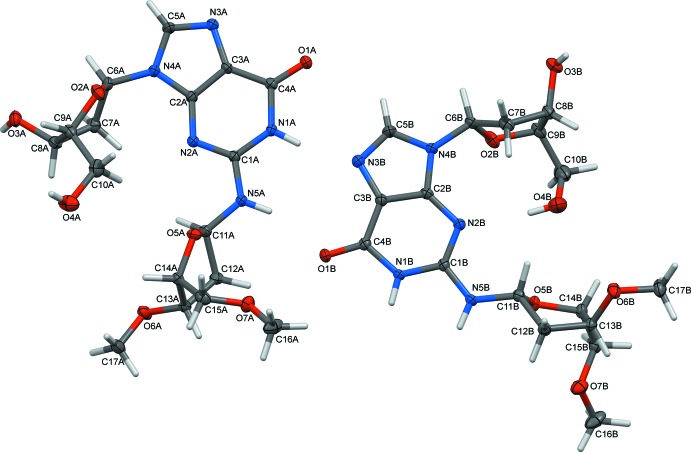
The mol­ecular structure of (I) showing 50% displacement ellipsoids.

**Figure 2:**
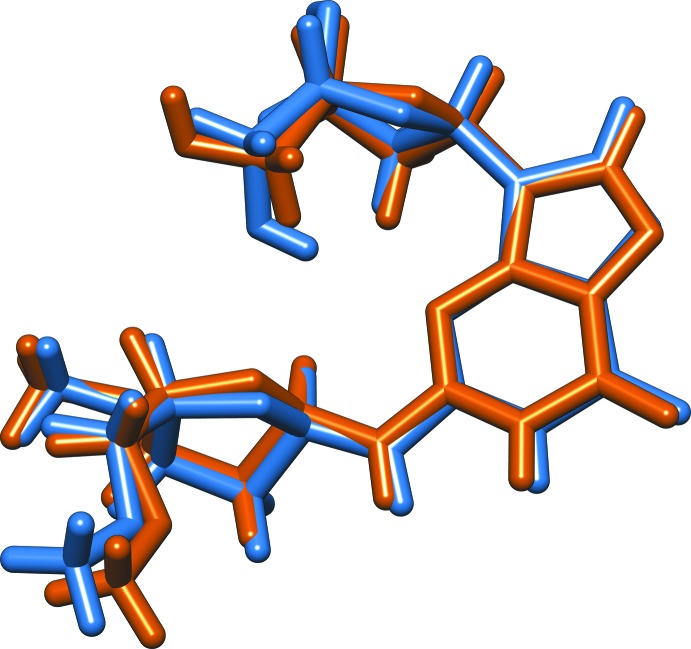
Overlay plot of the two mol­ecules in (I). *A* molecule in orange and *B* molecule in blue.

The two independent mol­ecules (*A* and *B*) of (I) are shown in Fig. 1 ▸ as they are oriented in the crystal, while Fig. 2 ▸ shows an overlay to illustrate the differences in orientation and conformation of the furan­ose rings. Ring puckering analysis, after Cremer & Pople as calculated using *PLATON* (Spek, 2009 ▸) indicates the furan­ose rings attached to N4 positions in the two mol­ecules to be half-chairs in both mol­ecules, but with the maximum variance from planarity occurring between C7 and C8 in mol­ecule *A* and C6 and C7 in mol­ecule *B* [*Q*(2) = 0.367 (2), Φ(2) = 88.0 (4)° for mol­ecule *A* and *Q*(2) = 0.347 (2), Φ(2) = 60.6 (4)° for mol­ecule *B*]. The disposition of these furan­ose rings relative to the purine rings can be described by the torsion angle C2—N4—C6—O2, which is 70.9 (3)° in mol­ecule *A* and 61.7 (3)° in mol­ecule *B*. The furan­ose ring attached to the N5 position in mol­ecule *A* is again a half-chair, with the maximum deviation from planarity between C11*A* and C12*A* [*Q*(2) = 3.41 (2), Φ(2) = 62.2 (3)°], while this furan­ose ring in mol­ecule *B* is an envelope with C11*B* at the flap [*Q*(2) = 0.422 (2), Φ(2) = 45.4 (3)°]. The disposition of these furan­ose rings relative to the purine rings can be described by the angle C1—N5—C11—O5, which is −87.4 (2)° in mol­ecule *A* and −93.7 (2)° in mol­ecule *B*.

## Supra­molecular features   

**Figure 3:**
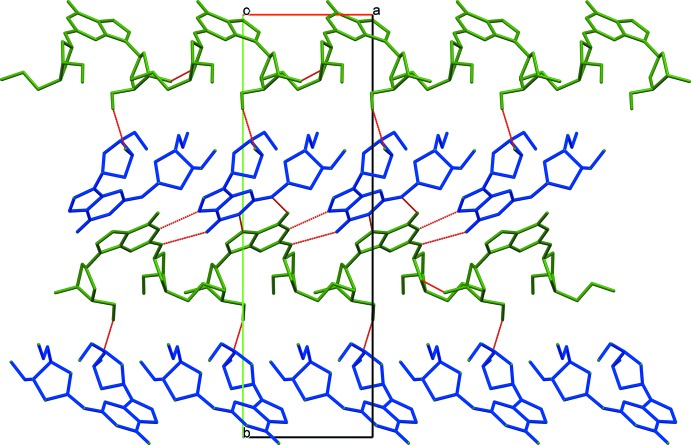
The packing in (I) along the *c* axis showing the formation of hydrogen-bonded chains (*A* mol­ecules green, *B* mol­ecules blue).

In the crystal, the two mol­ecules form infinite ribbons along the *a*–*c* diagonal of the unit cell, with the *A* mol­ecules on one side of the ribbon and the *B* mol­ecules on the other. The mol­ecules are staggered such that each *A* mol­ecule forms hydrogen bonds to two *B* mol­ecules and each *B* mol­ecule forms hydrogen bonds (Table 1 ▸) to two *A* mol­ecules, fully involving the N1, N3, N5 and O1 atoms. These ribbons are then stacked to form slabs propagating in the *ac* plane and one half the *b* dimension in thickness. The de­oxy­ribose moieties occupy the outsides of these slabs and are linked *via* hydrogen bonds to twofold screw-related slabs, resulting in a herringbone pattern in the three-dimensional structure as seen in Fig. 3 ▸.

## Database survey   

A search of the Cambridge Structural Database (CSD, Version 5.36, update February 2015; Groom & Allen, 2014 ▸) for de­oxy­guanosine analogues with exocyclic amine substitution revealed three crystal structures (Morr *et al.*, 1991 ▸; Fujino *et al.*, 2010 ▸). In all these crystal structures, the five-membered 2-de­oxy­ribo­furan­ose rings have envelope conformations, as in the title compound.

## Synthesis and crystallization   

2′-De­oxy­guanosine (199 mg, 0.75 mmol) and 3,5-bis-*O*-methyl-2-de­oxy-d-ribo­furan­ose (110 mg, 0.74 mmol) were dissolved in 0.8 ml of a 3:1 mixture of DMSO and 25 m*M* sodium phosphate buffer (pH 7.0) in a round-bottom flask. The flask was heated to 333 K and the mixture stirred for 22 h. The solvent removed *in vacuo* and the product purified by column chromatography on silica gel eluted with 0–15% methanol in di­chloro­methane (*R_f_* = 0.30, 15% methanol/di­chloro­methane) to yield 36 mg (12% yield) of the title compound as a colorless oil. The precursor 3,5-bis-*O*-methyl-2-de­oxy-d-ribo­furan­ose was synthesized according to previously reported procedures (Deriaz *et al.*, 1949 ▸; Olsson *et al.*, 1998 ▸). The title compound was crystallized by vapour diffusion, a 2 ml vial containing the title compound in methanol being placed in a 20 ml vial containing hexa­nes at room temperature for several days.

## Refinement   

Crystal data, data collection and structure refinement details are summarized in Table 2 ▸. H atoms were placed geometrically (C—H = 0.95 or 0.98 Å) and refined as riding with *U*_iso_(H) = 1.2*U*_eq_(C). 

## Supplementary Material

Crystal structure: contains datablock(s) I. DOI: 10.1107/S205698901600517X/hb7568sup1.cif

Structure factors: contains datablock(s) I. DOI: 10.1107/S205698901600517X/hb7568Isup2.hkl

CCDC reference: 1448235

Additional supporting information:  crystallographic information; 3D view; checkCIF report

## Figures and Tables

**Table 1:** Hydrogen-bond geometry (Å, °)

*D*—H⋯*A*	*D*—H	H⋯*A*	*D*⋯*A*	*D*—H⋯*A*
N1*A*—H1*A*⋯N3*B*	0.88	1.92	2.789 (2)	170
O3*A*—H3*A*⋯O5*A* ^i^	0.84	2.07	2.897 (2)	167
O4*A*—H4*A*⋯O3*B* ^ii^	0.84	2.01	2.847 (2)	178
N5*A*—H5*A*⋯O1*B*	0.88	2.23	3.058 (2)	157
C5*A*—H5*A*1⋯O1*B* ^iii^	0.95	2.63	3.284 (3)	126
C7*A*—H7*A*1⋯N2*A*	0.99	2.46	3.172 (3)	128
C8*A*—H8*A*⋯O7*A* ^i^	1.00	2.39	3.316 (3)	153
C12*A*—H12*A*⋯O1*A* ^iv^	0.99	2.61	3.432 (3)	141
C12*A*—H12*B*⋯O1*B*	0.99	2.55	3.426 (3)	147
C16*A*—H16*A*⋯O4*A* ^v^	0.98	2.47	3.401 (3)	158
C16*A*—H16*B*⋯O6*A* ^v^	0.98	2.54	3.222 (3)	127
C16*A*—H16*C*⋯O2*A* ^vi^	0.98	2.50	3.356 (3)	146
C17*A*—H17*A*⋯O3*A* ^vi^	0.98	2.65	3.610 (3)	168
C17*A*—H17*B*⋯O2*A* ^iv^	0.98	2.60	3.573 (3)	175
N1*B*—H1*B*⋯N3*A* ^vi^	0.88	1.94	2.808 (2)	166
O3*B*—H3*B*⋯O5*B* ^vii^	0.84	1.99	2.817 (2)	169
O4*B*—H4*B*⋯N2*B*	0.84	2.38	3.180 (3)	158
N5*B*—H5*B*⋯O1*A* ^vi^	0.88	2.19	3.027 (2)	159
C5*B*—H5*B*1⋯O1*A*	0.95	2.60	3.269 (3)	127
C8*B*—H8*B*⋯O7*B* ^vii^	1.00	2.49	3.363 (3)	146
C11*B*—H11*B*⋯O4*B*	1.00	2.59	3.251 (3)	124
C12*B*—H12*C*⋯O1*A* ^vi^	0.99	2.55	3.363 (3)	140
C12*B*—H12*D*⋯O1*B* ^v^	0.99	2.45	3.424 (3)	167
C14*B*—H14*B*⋯O4*B*	1.00	2.61	3.272 (3)	123
C17*B*—H17*E*⋯O2*B* ^v^	0.98	2.48	3.456 (3)	176

Symmetry codes: (i) 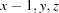; (ii) 
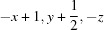
; (iii) 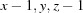; (iv) 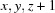; (v) 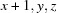; (vi) 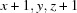; (vii) 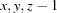.

**Table 2:** Experimental details

Crystal data	
Chemical formula	C_17_H_25_N_5_O_7_
*M*_r_	411.42
Crystal system, space group	Monoclinic, *P*2_1_
Temperature (K)	100
*a*, *b*, *c* (Å)	8.1817 (1), 26.4033 (5), 8.8800 (2)
β (°)	98.023 (1)
*V* (Å^3^)	1899.52 (6)
*Z*	4
Radiation type	Cu *K*α
μ (mm^−1^)	0.96
Crystal size (mm)	0.15 × 0.08 × 0.08

Data collection	
Diffractometer	Bruker APEXII CCD
Absorption correction	Multi-scan (*SADABS*; Sheldrick, 2008 ▸)
*T*_min_, *T*_max_	0.86, 0.93
No. of measured, independent and observed [*I* > 2σ(*I*)] reflections	26696, 6862, 6644
*R*_int_	0.029
(sin θ/λ)_max_ (Å^−1^)	0.617

Refinement	
*R*[*F* ^2^ > 2σ(*F* ^2^)], *wR*(*F* ^2^), *S*	0.027, 0.072, 1.04
No. of reflections	6862
No. of parameters	531
No. of restraints	1
H-atom treatment	H-atom parameters constrained
Δρ_max_, Δρ_min_ (e Å^−3^)	0.23, −0.17
Absolute structure	Flack *x* determined using 2923 quotients [(*I* ^+^)−(*I* ^−^)]/[(*I* ^+^)+(*I* ^−^)] (Parsons et al., 2013 ▸)
Absolute structure parameter	0.08 (5)

Computer programs: *APEX2* (Bruker, 2008 ▸), *SAINT* (Bruker, 2008 ▸), *SHELXS2013* (Sheldrick, 2008 ▸), *SHELXL2013* (Sheldrick, 2015 ▸), *X-SEED*, Barbour, 2001 ▸, *CIFTAB* (Sheldrick, 2008 ▸).

## supplementary crystallographic information

### Crystal data

**Table:**

C_17_H_25_N_5_O_7_	*F*(000) = 872
*M**_r_* = 411.42	*D*_x_ = 1.439 Mg m^−^^3^
Monoclinic, *P*2_1_	Cu *K*α radiation, λ = 1.54178 Å
*a* = 8.1817 (1) Å	Cell parameters from 9940 reflections
*b* = 26.4033 (5) Å	θ = 5.3–72.2°
*c* = 8.8800 (2) Å	µ = 0.96 mm^−^^1^
β = 98.023 (1)°	*T* = 100 K
*V* = 1899.52 (6) Å^3^	Prism, colourless
*Z* = 4	0.15 × 0.08 × 0.08 mm

### Data collection

**Table:**

Bruker APEXII CCD diffractometer	6644 reflections with *I* > 2σ(*I*)
Radiation source: Incoatec micro focus Cu tube	*R*_int_ = 0.029
ω and phi scans	θ_max_ = 72.2°, θ_min_ = 3.4°
Absorption correction: multi-scan (*SADABS*; Sheldrick, 2008)	*h* = −10→10
*T*_min_ = 0.86, *T*_max_ = 0.93	*k* = −31→31
26696 measured reflections	*l* = −9→10
6862 independent reflections	

### Refinement

**Table:**

Refinement on *F*^2^	Hydrogen site location: inferred from neighbouring sites
Least-squares matrix: full	H-atom parameters constrained
*R*[*F*^2^ > 2σ(*F*^2^)] = 0.027	*w* = 1/[σ^2^(*F*_o_^2^) + (0.0424*P*)^2^ + 0.3476*P*] where *P* = (*F*_o_^2^ + 2*F*_c_^2^)/3
*wR*(*F*^2^) = 0.072	(Δ/σ)_max_ < 0.001
*S* = 1.04	Δρ_max_ = 0.23 e Å^−^^3^
6862 reflections	Δρ_min_ = −0.17 e Å^−^^3^
531 parameters	Absolute structure: Flack *x* determined using 2923 quotients [(*I*^+^)-(*I*^-^)]/[(*I*^+^)+(*I*^-^)] (Parsons et al., 2013)
1 restraint	Absolute structure parameter: 0.08 (5)

### Special details

**Table:**

Geometry. All esds (except the esd in the dihedral angle between two l.s. planes) are estimated using the full covariance matrix. The cell esds are taken into account individually in the estimation of esds in distances, angles and torsion angles; correlations between esds in cell parameters are only used when they are defined by crystal symmetry. An approximate (isotropic) treatment of cell esds is used for estimating esds involving l.s. planes.

### Fractional atomic coordinates and isotropic or equivalent isotropic displacement parameters (Å^2^)

**Table:**

	*x*	*y*	*z*	*U*_iso_*/*U*_eq_	
O1A	0.34576 (17)	0.46915 (6)	−0.12995 (17)	0.0168 (3)	
N1A	0.3499 (2)	0.50765 (6)	0.1022 (2)	0.0146 (3)	
H1A	0.4493	0.4955	0.1317	0.017*	
C1A	0.2806 (2)	0.53738 (8)	0.2030 (2)	0.0140 (4)	
O2A	−0.1409 (2)	0.64849 (6)	0.01984 (18)	0.0223 (3)	
N2A	0.1339 (2)	0.55894 (7)	0.1729 (2)	0.0155 (4)	
C2A	0.0560 (3)	0.54690 (8)	0.0330 (2)	0.0150 (4)	
O3A	−0.44313 (19)	0.65756 (6)	0.18573 (19)	0.0221 (3)	
H3A	−0.5051	0.6460	0.2454	0.027*	
N3A	−0.0017 (2)	0.51309 (7)	−0.2041 (2)	0.0170 (4)	
C3A	0.1129 (2)	0.51624 (8)	−0.0744 (2)	0.0148 (4)	
O4A	0.0015 (2)	0.72195 (6)	0.3635 (2)	0.0298 (4)	
H4A	−0.0253	0.7501	0.3235	0.036*	
N4A	−0.0978 (2)	0.56295 (7)	−0.0324 (2)	0.0177 (4)	
C4A	0.2740 (2)	0.49535 (7)	−0.0439 (2)	0.0138 (4)	
O5A	0.37552 (18)	0.62783 (6)	0.42933 (16)	0.0176 (3)	
N5A	0.3711 (2)	0.54372 (7)	0.3418 (2)	0.0163 (4)	
H5A	0.4647	0.5272	0.3642	0.020*	
C5A	−0.1246 (3)	0.54152 (8)	−0.1750 (2)	0.0192 (4)	
H5A1	−0.2220	0.5468	−0.2449	0.023*	
O6A	0.23353 (18)	0.61868 (6)	0.75564 (18)	0.0227 (3)	
C6A	−0.2089 (3)	0.59897 (8)	0.0258 (3)	0.0185 (4)	
H6A	−0.3179	0.5980	−0.0410	0.022*	
O7A	0.69231 (18)	0.63615 (6)	0.60640 (19)	0.0221 (3)	
C7A	−0.2373 (3)	0.59060 (8)	0.1890 (3)	0.0184 (4)	
H7A1	−0.1372	0.5770	0.2514	0.022*	
H7A2	−0.3309	0.5673	0.1948	0.022*	
C8A	−0.2764 (3)	0.64379 (8)	0.2388 (2)	0.0182 (4)	
H8A	−0.2507	0.6476	0.3516	0.022*	
C9A	−0.1612 (3)	0.67599 (8)	0.1574 (2)	0.0189 (4)	
H9A	−0.2152	0.7093	0.1293	0.023*	
C10A	0.0085 (3)	0.68527 (9)	0.2482 (3)	0.0245 (5)	
H10A	0.0518	0.6531	0.2951	0.029*	
H10B	0.0853	0.6970	0.1787	0.029*	
C11A	0.3171 (2)	0.57690 (8)	0.4527 (2)	0.0156 (4)	
H11A	0.1938	0.5767	0.4429	0.019*	
C12A	0.3901 (3)	0.56396 (8)	0.6145 (2)	0.0182 (4)	
H12A	0.3228	0.5381	0.6581	0.022*	
H12B	0.5045	0.5513	0.6190	0.022*	
C13A	0.3861 (2)	0.61397 (8)	0.6978 (2)	0.0167 (4)	
H13A	0.4804	0.6161	0.7823	0.020*	
C14A	0.4064 (3)	0.65359 (8)	0.5738 (2)	0.0175 (4)	
H14A	0.3230	0.6811	0.5769	0.021*	
C15A	0.5775 (3)	0.67638 (9)	0.5938 (3)	0.0218 (4)	
H15A	0.5917	0.6979	0.5053	0.026*	
H15B	0.5947	0.6976	0.6866	0.026*	
C16A	0.8590 (3)	0.65335 (11)	0.6422 (3)	0.0301 (5)	
H16A	0.8799	0.6802	0.5711	0.045*	
H16B	0.9346	0.6250	0.6338	0.045*	
H16C	0.8766	0.6666	0.7463	0.045*	
C17A	0.2364 (3)	0.65893 (11)	0.8622 (3)	0.0289 (5)	
H17A	0.3324	0.6551	0.9407	0.043*	
H17B	0.1352	0.6581	0.9096	0.043*	
H17C	0.2435	0.6913	0.8097	0.043*	
O1B	0.72354 (17)	0.51384 (5)	0.47267 (17)	0.0168 (3)	
N1B	0.9716 (2)	0.47201 (6)	0.50184 (19)	0.0139 (3)	
H1B	0.9971	0.4855	0.5927	0.017*	
C1B	1.0833 (2)	0.43911 (7)	0.4530 (2)	0.0136 (4)	
O2B	0.9310 (2)	0.32766 (6)	0.07593 (19)	0.0219 (3)	
N2B	1.0610 (2)	0.41620 (6)	0.3193 (2)	0.0153 (4)	
C2B	0.9168 (3)	0.42949 (8)	0.2340 (2)	0.0150 (4)	
O3B	1.09413 (19)	0.31769 (6)	−0.23244 (18)	0.0220 (3)	
H3B	1.1571	0.3306	−0.2890	0.026*	
N3B	0.6634 (2)	0.46447 (7)	0.1606 (2)	0.0188 (4)	
C3B	0.7979 (2)	0.46230 (8)	0.2729 (2)	0.0159 (4)	
O4B	1.2568 (3)	0.31747 (9)	0.2436 (2)	0.0452 (5)	
H4B	1.1920	0.3377	0.2779	0.054*	
N4B	0.8528 (2)	0.41146 (7)	0.0918 (2)	0.0186 (4)	
C4B	0.8210 (2)	0.48559 (8)	0.4184 (2)	0.0143 (4)	
O5B	1.32491 (17)	0.34807 (6)	0.57774 (18)	0.0175 (3)	
N5B	1.2221 (2)	0.43116 (7)	0.5529 (2)	0.0159 (3)	
H5B	1.2334	0.4468	0.6413	0.019*	
C5B	0.7002 (3)	0.43335 (9)	0.0558 (3)	0.0215 (5)	
H5B1	0.6287	0.4266	−0.0358	0.026*	
O6B	1.67925 (19)	0.36348 (6)	0.43876 (18)	0.0216 (3)	
C6B	0.9187 (3)	0.37405 (8)	−0.0034 (2)	0.0185 (4)	
H6B	0.8402	0.3699	−0.0995	0.022*	
O7B	1.5367 (2)	0.31343 (6)	0.84948 (19)	0.0252 (3)	
C7B	1.0893 (3)	0.38469 (8)	−0.0431 (3)	0.0198 (4)	
H7B1	1.1615	0.4002	0.0435	0.024*	
H7B2	1.0845	0.4072	−0.1328	0.024*	
C8B	1.1484 (3)	0.33176 (8)	−0.0781 (2)	0.0191 (4)	
H8B	1.2709	0.3287	−0.0525	0.023*	
C9B	1.0581 (3)	0.29760 (9)	0.0248 (3)	0.0226 (5)	
H9B	1.0071	0.2683	−0.0357	0.027*	
C10B	1.1657 (4)	0.27835 (11)	0.1641 (3)	0.0360 (6)	
H10C	1.0958	0.2618	0.2322	0.043*	
H10D	1.2427	0.2526	0.1337	0.043*	
C11B	1.3507 (3)	0.39806 (8)	0.5188 (2)	0.0157 (4)	
H11B	1.3500	0.3963	0.4062	0.019*	
C12B	1.5222 (3)	0.41144 (8)	0.5959 (3)	0.0191 (4)	
H12C	1.5218	0.4204	0.7041	0.023*	
H12D	1.5700	0.4398	0.5436	0.023*	
C13B	1.6149 (3)	0.36211 (8)	0.5792 (2)	0.0177 (4)	
H13B	1.7050	0.3572	0.6664	0.021*	
C14B	1.4792 (3)	0.32117 (8)	0.5795 (3)	0.0178 (4)	
H14B	1.4738	0.3009	0.4838	0.021*	
C15B	1.5051 (3)	0.28550 (9)	0.7128 (3)	0.0227 (5)	
H15C	1.4055	0.2643	0.7142	0.027*	
H15D	1.5995	0.2628	0.7033	0.027*	
C16B	1.5735 (3)	0.28046 (11)	0.9765 (3)	0.0331 (6)	
H16D	1.4856	0.2552	0.9752	0.050*	
H16E	1.5814	0.3002	1.0708	0.050*	
H16F	1.6788	0.2633	0.9711	0.050*	
C17B	1.7854 (3)	0.32160 (10)	0.4226 (3)	0.0271 (5)	
H17D	1.8756	0.3212	0.5077	0.041*	
H17E	1.8312	0.3248	0.3267	0.041*	
H17F	1.7225	0.2900	0.4221	0.041*	

### Atomic displacement parameters (Å^2^)

**Table:**

	*U*^11^	*U*^22^	*U*^33^	*U*^12^	*U*^13^	*U*^23^
O1A	0.0171 (7)	0.0182 (7)	0.0155 (7)	0.0035 (6)	0.0033 (5)	−0.0025 (6)
N1A	0.0120 (7)	0.0159 (8)	0.0155 (9)	0.0028 (6)	0.0006 (6)	−0.0015 (6)
C1A	0.0148 (9)	0.0128 (9)	0.0142 (10)	−0.0002 (7)	0.0018 (7)	−0.0013 (7)
O2A	0.0304 (9)	0.0195 (8)	0.0191 (8)	0.0039 (6)	0.0108 (6)	0.0007 (6)
N2A	0.0152 (8)	0.0175 (9)	0.0134 (9)	0.0031 (6)	0.0008 (7)	−0.0026 (7)
C2A	0.0152 (9)	0.0136 (10)	0.0158 (10)	0.0012 (7)	0.0005 (7)	−0.0019 (7)
O3A	0.0198 (8)	0.0247 (8)	0.0234 (8)	0.0066 (6)	0.0078 (6)	0.0027 (6)
N3A	0.0174 (8)	0.0183 (9)	0.0148 (9)	0.0033 (7)	0.0001 (7)	−0.0032 (7)
C3A	0.0170 (9)	0.0137 (9)	0.0134 (10)	0.0018 (8)	0.0014 (8)	−0.0032 (7)
O4A	0.0448 (10)	0.0194 (8)	0.0243 (9)	−0.0047 (7)	0.0011 (7)	0.0001 (7)
N4A	0.0172 (8)	0.0195 (9)	0.0154 (9)	0.0053 (7)	−0.0012 (7)	−0.0036 (7)
C4A	0.0152 (10)	0.0127 (10)	0.0139 (10)	−0.0012 (7)	0.0032 (8)	−0.0005 (7)
O5A	0.0227 (7)	0.0174 (7)	0.0123 (7)	−0.0008 (6)	0.0009 (5)	−0.0013 (6)
N5A	0.0139 (8)	0.0201 (9)	0.0142 (9)	0.0039 (6)	−0.0003 (6)	−0.0034 (7)
C5A	0.0169 (10)	0.0228 (11)	0.0166 (11)	0.0045 (8)	−0.0027 (8)	−0.0048 (8)
O6A	0.0163 (7)	0.0333 (9)	0.0194 (8)	−0.0025 (6)	0.0060 (6)	−0.0082 (7)
C6A	0.0163 (9)	0.0200 (11)	0.0185 (11)	0.0069 (8)	0.0005 (8)	−0.0024 (8)
O7A	0.0153 (7)	0.0240 (8)	0.0268 (9)	−0.0015 (6)	0.0027 (6)	0.0011 (6)
C7A	0.0172 (10)	0.0172 (11)	0.0211 (11)	0.0001 (8)	0.0037 (8)	0.0002 (8)
C8A	0.0203 (10)	0.0201 (11)	0.0148 (10)	0.0015 (8)	0.0047 (8)	−0.0008 (8)
C9A	0.0257 (11)	0.0157 (10)	0.0165 (10)	0.0038 (8)	0.0073 (8)	0.0009 (8)
C10A	0.0259 (12)	0.0182 (11)	0.0297 (13)	0.0001 (9)	0.0052 (10)	0.0023 (9)
C11A	0.0150 (9)	0.0167 (10)	0.0149 (10)	0.0005 (7)	0.0015 (7)	−0.0028 (8)
C12A	0.0206 (10)	0.0201 (10)	0.0137 (10)	0.0017 (8)	0.0024 (8)	−0.0002 (8)
C13A	0.0139 (9)	0.0227 (11)	0.0134 (10)	0.0007 (8)	0.0012 (7)	−0.0029 (8)
C14A	0.0185 (10)	0.0172 (10)	0.0163 (10)	0.0037 (8)	0.0004 (8)	−0.0042 (8)
C15A	0.0257 (11)	0.0193 (11)	0.0207 (11)	−0.0025 (9)	0.0043 (9)	−0.0023 (9)
C16A	0.0209 (11)	0.0458 (15)	0.0230 (12)	−0.0085 (10)	0.0010 (9)	0.0059 (11)
C17A	0.0229 (11)	0.0410 (14)	0.0238 (12)	0.0011 (10)	0.0065 (9)	−0.0138 (10)
O1B	0.0172 (7)	0.0172 (7)	0.0163 (7)	0.0052 (6)	0.0031 (6)	−0.0026 (6)
N1B	0.0149 (8)	0.0147 (8)	0.0119 (8)	0.0022 (6)	0.0009 (6)	−0.0038 (6)
C1B	0.0148 (9)	0.0122 (9)	0.0140 (10)	0.0001 (7)	0.0033 (7)	0.0005 (7)
O2B	0.0261 (8)	0.0176 (8)	0.0243 (8)	−0.0016 (6)	0.0113 (6)	−0.0048 (6)
N2B	0.0137 (8)	0.0171 (9)	0.0148 (9)	0.0019 (6)	0.0010 (6)	−0.0028 (7)
C2B	0.0169 (9)	0.0146 (10)	0.0136 (10)	−0.0007 (8)	0.0022 (7)	−0.0021 (8)
O3B	0.0278 (8)	0.0215 (8)	0.0183 (8)	−0.0035 (6)	0.0094 (6)	−0.0033 (6)
N3B	0.0168 (8)	0.0222 (9)	0.0166 (9)	0.0042 (7)	−0.0003 (7)	−0.0038 (7)
C3B	0.0144 (9)	0.0162 (10)	0.0168 (10)	0.0017 (8)	0.0014 (8)	−0.0016 (8)
O4B	0.0386 (11)	0.0665 (15)	0.0278 (10)	0.0170 (10)	−0.0056 (8)	−0.0101 (10)
N4B	0.0168 (9)	0.0227 (9)	0.0153 (9)	0.0048 (7)	−0.0013 (7)	−0.0068 (7)
C4B	0.0154 (9)	0.0128 (9)	0.0147 (10)	−0.0004 (7)	0.0024 (7)	0.0014 (7)
O5B	0.0139 (7)	0.0165 (7)	0.0226 (8)	0.0023 (6)	0.0037 (6)	0.0013 (6)
N5B	0.0172 (8)	0.0162 (8)	0.0137 (8)	0.0038 (7)	0.0004 (6)	−0.0042 (6)
C5B	0.0185 (10)	0.0270 (12)	0.0174 (11)	0.0039 (9)	−0.0028 (8)	−0.0053 (9)
O6B	0.0225 (8)	0.0239 (8)	0.0200 (8)	0.0048 (6)	0.0084 (6)	0.0045 (6)
C6B	0.0212 (11)	0.0186 (11)	0.0151 (10)	0.0027 (8)	0.0002 (8)	−0.0061 (8)
O7B	0.0264 (8)	0.0281 (9)	0.0207 (8)	0.0003 (7)	0.0018 (6)	0.0081 (7)
C7B	0.0246 (11)	0.0175 (10)	0.0181 (11)	−0.0018 (8)	0.0055 (8)	−0.0025 (8)
C8B	0.0204 (10)	0.0202 (11)	0.0175 (11)	0.0020 (8)	0.0054 (8)	−0.0025 (8)
C9B	0.0305 (12)	0.0183 (11)	0.0208 (11)	0.0022 (9)	0.0098 (9)	−0.0036 (8)
C10B	0.0506 (16)	0.0338 (14)	0.0241 (13)	0.0162 (12)	0.0067 (11)	0.0026 (11)
C11B	0.0174 (10)	0.0146 (10)	0.0151 (10)	0.0013 (7)	0.0018 (8)	0.0003 (7)
C12B	0.0156 (10)	0.0195 (11)	0.0220 (11)	0.0002 (8)	0.0023 (8)	−0.0036 (8)
C13B	0.0149 (10)	0.0209 (11)	0.0174 (11)	0.0028 (8)	0.0028 (8)	0.0004 (8)
C14B	0.0160 (9)	0.0167 (10)	0.0210 (11)	0.0035 (8)	0.0040 (8)	−0.0011 (8)
C15B	0.0201 (10)	0.0200 (11)	0.0280 (12)	0.0021 (8)	0.0034 (9)	0.0031 (9)
C16B	0.0261 (12)	0.0417 (15)	0.0298 (13)	−0.0035 (11)	−0.0019 (10)	0.0187 (11)
C17B	0.0263 (12)	0.0291 (13)	0.0281 (12)	0.0092 (10)	0.0118 (9)	0.0021 (10)

### Geometric parameters (Å, º)

**Table:**

O1A—C4A	1.238 (3)	O1B—C4B	1.237 (3)
N1A—C1A	1.371 (3)	N1B—C1B	1.374 (3)
N1A—C4A	1.397 (3)	N1B—C4B	1.393 (3)
N1A—H1A	0.8800	N1B—H1B	0.8800
C1A—N2A	1.321 (3)	C1B—N2B	1.323 (3)
C1A—N5A	1.357 (3)	C1B—N5B	1.356 (3)
O2A—C6A	1.425 (3)	O2B—C6B	1.409 (3)
O2A—C9A	1.450 (3)	O2B—C9B	1.432 (3)
N2A—C2A	1.353 (3)	N2B—C2B	1.356 (3)
C2A—N4A	1.377 (3)	C2B—C3B	1.382 (3)
C2A—C3A	1.380 (3)	C2B—N4B	1.382 (3)
O3A—C8A	1.427 (3)	O3B—C8B	1.429 (3)
O3A—H3A	0.8400	O3B—H3B	0.8400
N3A—C5A	1.309 (3)	N3B—C5B	1.308 (3)
N3A—C3A	1.382 (3)	N3B—C3B	1.379 (3)
C3A—C4A	1.420 (3)	C3B—C4B	1.420 (3)
O4A—C10A	1.417 (3)	O4B—C10B	1.405 (4)
O4A—H4A	0.8400	O4B—H4B	0.8400
N4A—C5A	1.376 (3)	N4B—C5B	1.373 (3)
N4A—C6A	1.460 (3)	N4B—C6B	1.452 (3)
O5A—C14A	1.443 (2)	O5B—C11B	1.446 (3)
O5A—C11A	1.452 (3)	O5B—C14B	1.447 (2)
N5A—C11A	1.433 (3)	N5B—C11B	1.433 (3)
N5A—H5A	0.8800	N5B—H5B	0.8800
C5A—H5A1	0.9500	C5B—H5B1	0.9500
O6A—C13A	1.420 (3)	O6B—C13B	1.420 (3)
O6A—C17A	1.421 (3)	O6B—C17B	1.426 (3)
C6A—C7A	1.515 (3)	C6B—C7B	1.513 (3)
C6A—H6A	1.0000	C6B—H6B	1.0000
O7A—C15A	1.412 (3)	O7B—C15B	1.413 (3)
O7A—C16A	1.431 (3)	O7B—C16B	1.424 (3)
C7A—C8A	1.520 (3)	C7B—C8B	1.525 (3)
C7A—H7A1	0.9900	C7B—H7B1	0.9900
C7A—H7A2	0.9900	C7B—H7B2	0.9900
C8A—C9A	1.524 (3)	C8B—C9B	1.544 (3)
C8A—H8A	1.0000	C8B—H8B	1.0000
C9A—C10A	1.525 (3)	C9B—C10B	1.503 (4)
C9A—H9A	1.0000	C9B—H9B	1.0000
C10A—H10A	0.9900	C10B—H10C	0.9900
C10A—H10B	0.9900	C10B—H10D	0.9900
C11A—C12A	1.516 (3)	C11B—C12B	1.514 (3)
C11A—H11A	1.0000	C11B—H11B	1.0000
C12A—C13A	1.516 (3)	C12B—C13B	1.525 (3)
C12A—H12A	0.9900	C12B—H12C	0.9900
C12A—H12B	0.9900	C12B—H12D	0.9900
C13A—C14A	1.544 (3)	C13B—C14B	1.550 (3)
C13A—H13A	1.0000	C13B—H13B	1.0000
C14A—C15A	1.512 (3)	C14B—C15B	1.505 (3)
C14A—H14A	1.0000	C14B—H14B	1.0000
C15A—H15A	0.9900	C15B—H15C	0.9900
C15A—H15B	0.9900	C15B—H15D	0.9900
C16A—H16A	0.9800	C16B—H16D	0.9800
C16A—H16B	0.9800	C16B—H16E	0.9800
C16A—H16C	0.9800	C16B—H16F	0.9800
C17A—H17A	0.9800	C17B—H17D	0.9800
C17A—H17B	0.9800	C17B—H17E	0.9800
C17A—H17C	0.9800	C17B—H17F	0.9800
			
C1A—N1A—C4A	124.64 (17)	C1B—N1B—C4B	124.95 (17)
C1A—N1A—H1A	117.7	C1B—N1B—H1B	117.5
C4A—N1A—H1A	117.7	C4B—N1B—H1B	117.5
N2A—C1A—N5A	119.73 (18)	N2B—C1B—N5B	120.96 (18)
N2A—C1A—N1A	124.14 (18)	N2B—C1B—N1B	123.93 (18)
N5A—C1A—N1A	116.13 (17)	N5B—C1B—N1B	115.11 (18)
C6A—O2A—C9A	109.66 (16)	C6B—O2B—C9B	109.15 (17)
C1A—N2A—C2A	112.51 (17)	C1B—N2B—C2B	112.52 (17)
N2A—C2A—N4A	126.92 (19)	N2B—C2B—C3B	127.64 (19)
N2A—C2A—C3A	127.70 (19)	N2B—C2B—N4B	127.58 (19)
N4A—C2A—C3A	105.38 (18)	C3B—C2B—N4B	104.73 (18)
C8A—O3A—H3A	109.5	C8B—O3B—H3B	109.5
C5A—N3A—C3A	104.51 (18)	C5B—N3B—C3B	104.43 (17)
C2A—C3A—N3A	110.89 (18)	N3B—C3B—C2B	111.34 (19)
C2A—C3A—C4A	119.30 (18)	N3B—C3B—C4B	129.16 (19)
N3A—C3A—C4A	129.73 (19)	C2B—C3B—C4B	119.33 (19)
C10A—O4A—H4A	109.5	C10B—O4B—H4B	109.5
C5A—N4A—C2A	106.28 (17)	C5B—N4B—C2B	106.50 (17)
C5A—N4A—C6A	124.53 (18)	C5B—N4B—C6B	123.40 (18)
C2A—N4A—C6A	128.96 (18)	C2B—N4B—C6B	129.95 (18)
O1A—C4A—N1A	121.03 (18)	O1B—C4B—N1B	121.23 (19)
O1A—C4A—C3A	127.38 (19)	O1B—C4B—C3B	127.16 (19)
N1A—C4A—C3A	111.59 (17)	N1B—C4B—C3B	111.59 (17)
C14A—O5A—C11A	109.25 (15)	C11B—O5B—C14B	106.31 (15)
C1A—N5A—C11A	121.20 (17)	C1B—N5B—C11B	121.92 (18)
C1A—N5A—H5A	119.4	C1B—N5B—H5B	119.0
C11A—N5A—H5A	119.4	C11B—N5B—H5B	119.0
N3A—C5A—N4A	112.93 (18)	N3B—C5B—N4B	112.99 (18)
N3A—C5A—H5A1	123.5	N3B—C5B—H5B1	123.5
N4A—C5A—H5A1	123.5	N4B—C5B—H5B1	123.5
C13A—O6A—C17A	111.90 (16)	C13B—O6B—C17B	112.00 (17)
O2A—C6A—N4A	108.59 (17)	O2B—C6B—N4B	107.89 (18)
O2A—C6A—C7A	106.39 (17)	O2B—C6B—C7B	105.90 (17)
N4A—C6A—C7A	115.46 (18)	N4B—C6B—C7B	116.03 (18)
O2A—C6A—H6A	108.7	O2B—C6B—H6B	108.9
N4A—C6A—H6A	108.7	N4B—C6B—H6B	108.9
C7A—C6A—H6A	108.7	C7B—C6B—H6B	108.9
C15A—O7A—C16A	112.43 (18)	C15B—O7B—C16B	110.76 (19)
C6A—C7A—C8A	102.12 (17)	C6B—C7B—C8B	101.90 (17)
C6A—C7A—H7A1	111.3	C6B—C7B—H7B1	111.4
C8A—C7A—H7A1	111.3	C8B—C7B—H7B1	111.4
C6A—C7A—H7A2	111.3	C6B—C7B—H7B2	111.4
C8A—C7A—H7A2	111.3	C8B—C7B—H7B2	111.4
H7A1—C7A—H7A2	109.2	H7B1—C7B—H7B2	109.3
O3A—C8A—C7A	111.70 (18)	O3B—C8B—C7B	111.64 (18)
O3A—C8A—C9A	109.09 (17)	O3B—C8B—C9B	107.79 (17)
C7A—C8A—C9A	102.03 (17)	C7B—C8B—C9B	102.92 (17)
O3A—C8A—H8A	111.2	O3B—C8B—H8B	111.4
C7A—C8A—H8A	111.2	C7B—C8B—H8B	111.4
C9A—C8A—H8A	111.2	C9B—C8B—H8B	111.4
O2A—C9A—C8A	105.75 (17)	O2B—C9B—C10B	107.12 (19)
O2A—C9A—C10A	108.82 (17)	O2B—C9B—C8B	107.00 (17)
C8A—C9A—C10A	114.59 (18)	C10B—C9B—C8B	114.3 (2)
O2A—C9A—H9A	109.2	O2B—C9B—H9B	109.4
C8A—C9A—H9A	109.2	C10B—C9B—H9B	109.4
C10A—C9A—H9A	109.2	C8B—C9B—H9B	109.4
O4A—C10A—C9A	111.51 (19)	O4B—C10B—C9B	111.9 (2)
O4A—C10A—H10A	109.3	O4B—C10B—H10C	109.2
C9A—C10A—H10A	109.3	C9B—C10B—H10C	109.2
O4A—C10A—H10B	109.3	O4B—C10B—H10D	109.2
C9A—C10A—H10B	109.3	C9B—C10B—H10D	109.2
H10A—C10A—H10B	108.0	H10C—C10B—H10D	107.9
N5A—C11A—O5A	109.19 (17)	N5B—C11B—O5B	109.36 (17)
N5A—C11A—C12A	113.26 (17)	N5B—C11B—C12B	115.06 (18)
O5A—C11A—C12A	104.50 (16)	O5B—C11B—C12B	102.84 (16)
N5A—C11A—H11A	109.9	N5B—C11B—H11B	109.8
O5A—C11A—H11A	109.9	O5B—C11B—H11B	109.8
C12A—C11A—H11A	109.9	C12B—C11B—H11B	109.8
C13A—C12A—C11A	103.50 (17)	C11B—C12B—C13B	101.47 (17)
C13A—C12A—H12A	111.1	C11B—C12B—H12C	111.5
C11A—C12A—H12A	111.1	C13B—C12B—H12C	111.5
C13A—C12A—H12B	111.1	C11B—C12B—H12D	111.5
C11A—C12A—H12B	111.1	C13B—C12B—H12D	111.5
H12A—C12A—H12B	109.0	H12C—C12B—H12D	109.3
O6A—C13A—C12A	109.43 (17)	O6B—C13B—C12B	108.29 (17)
O6A—C13A—C14A	112.71 (17)	O6B—C13B—C14B	111.87 (18)
C12A—C13A—C14A	103.35 (16)	C12B—C13B—C14B	103.26 (16)
O6A—C13A—H13A	110.4	O6B—C13B—H13B	111.0
C12A—C13A—H13A	110.4	C12B—C13B—H13B	111.0
C14A—C13A—H13A	110.4	C14B—C13B—H13B	111.0
O5A—C14A—C15A	109.66 (17)	O5B—C14B—C15B	110.05 (17)
O5A—C14A—C13A	106.95 (16)	O5B—C14B—C13B	106.37 (16)
C15A—C14A—C13A	112.09 (17)	C15B—C14B—C13B	114.55 (18)
O5A—C14A—H14A	109.4	O5B—C14B—H14B	108.6
C15A—C14A—H14A	109.4	C15B—C14B—H14B	108.6
C13A—C14A—H14A	109.4	C13B—C14B—H14B	108.6
O7A—C15A—C14A	107.74 (17)	O7B—C15B—C14B	109.74 (18)
O7A—C15A—H15A	110.2	O7B—C15B—H15C	109.7
C14A—C15A—H15A	110.2	C14B—C15B—H15C	109.7
O7A—C15A—H15B	110.2	O7B—C15B—H15D	109.7
C14A—C15A—H15B	110.2	C14B—C15B—H15D	109.7
H15A—C15A—H15B	108.5	H15C—C15B—H15D	108.2
O7A—C16A—H16A	109.5	O7B—C16B—H16D	109.5
O7A—C16A—H16B	109.5	O7B—C16B—H16E	109.5
H16A—C16A—H16B	109.5	H16D—C16B—H16E	109.5
O7A—C16A—H16C	109.5	O7B—C16B—H16F	109.5
H16A—C16A—H16C	109.5	H16D—C16B—H16F	109.5
H16B—C16A—H16C	109.5	H16E—C16B—H16F	109.5
O6A—C17A—H17A	109.5	O6B—C17B—H17D	109.5
O6A—C17A—H17B	109.5	O6B—C17B—H17E	109.5
H17A—C17A—H17B	109.5	H17D—C17B—H17E	109.5
O6A—C17A—H17C	109.5	O6B—C17B—H17F	109.5
H17A—C17A—H17C	109.5	H17D—C17B—H17F	109.5
H17B—C17A—H17C	109.5	H17E—C17B—H17F	109.5
			
C4A—N1A—C1A—N2A	−1.4 (3)	C4B—N1B—C1B—N2B	0.2 (3)
C4A—N1A—C1A—N5A	177.84 (18)	C4B—N1B—C1B—N5B	−179.83 (18)
N5A—C1A—N2A—C2A	−176.82 (19)	N5B—C1B—N2B—C2B	−178.87 (19)
N1A—C1A—N2A—C2A	2.4 (3)	N1B—C1B—N2B—C2B	1.1 (3)
C1A—N2A—C2A—N4A	179.8 (2)	C1B—N2B—C2B—C3B	−0.8 (3)
C1A—N2A—C2A—C3A	−0.3 (3)	C1B—N2B—C2B—N4B	−177.6 (2)
N2A—C2A—C3A—N3A	−179.8 (2)	C5B—N3B—C3B—C2B	0.2 (3)
N4A—C2A—C3A—N3A	0.1 (2)	C5B—N3B—C3B—C4B	−175.1 (2)
N2A—C2A—C3A—C4A	−2.9 (3)	N2B—C2B—C3B—N3B	−176.7 (2)
N4A—C2A—C3A—C4A	177.03 (18)	N4B—C2B—C3B—N3B	0.7 (2)
C5A—N3A—C3A—C2A	0.2 (2)	N2B—C2B—C3B—C4B	−0.9 (3)
C5A—N3A—C3A—C4A	−176.3 (2)	N4B—C2B—C3B—C4B	176.47 (19)
N2A—C2A—N4A—C5A	179.5 (2)	N2B—C2B—N4B—C5B	176.1 (2)
C3A—C2A—N4A—C5A	−0.4 (2)	C3B—C2B—N4B—C5B	−1.3 (2)
N2A—C2A—N4A—C6A	4.8 (4)	N2B—C2B—N4B—C6B	0.6 (4)
C3A—C2A—N4A—C6A	−175.1 (2)	C3B—C2B—N4B—C6B	−176.8 (2)
C1A—N1A—C4A—O1A	178.41 (19)	C1B—N1B—C4B—O1B	176.86 (19)
C1A—N1A—C4A—C3A	−1.7 (3)	C1B—N1B—C4B—C3B	−1.8 (3)
C2A—C3A—C4A—O1A	−176.6 (2)	N3B—C3B—C4B—O1B	−1.6 (4)
N3A—C3A—C4A—O1A	−0.3 (4)	C2B—C3B—C4B—O1B	−176.5 (2)
C2A—C3A—C4A—N1A	3.6 (3)	N3B—C3B—C4B—N1B	177.0 (2)
N3A—C3A—C4A—N1A	179.8 (2)	C2B—C3B—C4B—N1B	2.0 (3)
N2A—C1A—N5A—C11A	−5.3 (3)	N2B—C1B—N5B—C11B	0.3 (3)
N1A—C1A—N5A—C11A	175.39 (18)	N1B—C1B—N5B—C11B	−179.74 (18)
C3A—N3A—C5A—N4A	−0.5 (3)	C3B—N3B—C5B—N4B	−1.0 (3)
C2A—N4A—C5A—N3A	0.6 (3)	C2B—N4B—C5B—N3B	1.5 (3)
C6A—N4A—C5A—N3A	175.6 (2)	C6B—N4B—C5B—N3B	177.4 (2)
C9A—O2A—C6A—N4A	−138.45 (17)	C9B—O2B—C6B—N4B	−153.74 (17)
C9A—O2A—C6A—C7A	−13.6 (2)	C9B—O2B—C6B—C7B	−28.9 (2)
C5A—N4A—C6A—O2A	−103.0 (2)	C5B—N4B—C6B—O2B	−113.2 (2)
C2A—N4A—C6A—O2A	70.9 (3)	C2B—N4B—C6B—O2B	61.7 (3)
C5A—N4A—C6A—C7A	137.7 (2)	C5B—N4B—C6B—C7B	128.3 (2)
C2A—N4A—C6A—C7A	−48.4 (3)	C2B—N4B—C6B—C7B	−56.9 (3)
O2A—C6A—C7A—C8A	31.8 (2)	O2B—C6B—C7B—C8B	36.8 (2)
N4A—C6A—C7A—C8A	152.34 (18)	N4B—C6B—C7B—C8B	156.41 (19)
C6A—C7A—C8A—O3A	79.6 (2)	C6B—C7B—C8B—O3B	85.4 (2)
C6A—C7A—C8A—C9A	−36.8 (2)	C6B—C7B—C8B—C9B	−30.0 (2)
C6A—O2A—C9A—C8A	−10.4 (2)	C6B—O2B—C9B—C10B	131.8 (2)
C6A—O2A—C9A—C10A	113.18 (18)	C6B—O2B—C9B—C8B	8.8 (2)
O3A—C8A—C9A—O2A	−88.60 (19)	O3B—C8B—C9B—O2B	−103.77 (19)
C7A—C8A—C9A—O2A	29.7 (2)	C7B—C8B—C9B—O2B	14.3 (2)
O3A—C8A—C9A—C10A	151.55 (18)	O3B—C8B—C9B—C10B	137.8 (2)
C7A—C8A—C9A—C10A	−90.2 (2)	C7B—C8B—C9B—C10B	−104.1 (2)
O2A—C9A—C10A—O4A	165.83 (17)	O2B—C9B—C10B—O4B	−68.9 (3)
C8A—C9A—C10A—O4A	−76.0 (2)	C8B—C9B—C10B—O4B	49.4 (3)
C1A—N5A—C11A—O5A	−87.4 (2)	C1B—N5B—C11B—O5B	−93.7 (2)
C1A—N5A—C11A—C12A	156.64 (19)	C1B—N5B—C11B—C12B	151.2 (2)
C14A—O5A—C11A—N5A	−148.25 (16)	C14B—O5B—C11B—N5B	−163.87 (16)
C14A—O5A—C11A—C12A	−26.8 (2)	C14B—O5B—C11B—C12B	−41.2 (2)
N5A—C11A—C12A—C13A	154.32 (17)	N5B—C11B—C12B—C13B	162.67 (18)
O5A—C11A—C12A—C13A	35.6 (2)	O5B—C11B—C12B—C13B	43.9 (2)
C17A—O6A—C13A—C12A	167.04 (19)	C17B—O6B—C13B—C12B	172.60 (18)
C17A—O6A—C13A—C14A	−78.6 (2)	C17B—O6B—C13B—C14B	−74.3 (2)
C11A—C12A—C13A—O6A	89.7 (2)	C11B—C12B—C13B—O6B	88.8 (2)
C11A—C12A—C13A—C14A	−30.6 (2)	C11B—C12B—C13B—C14B	−30.0 (2)
C11A—O5A—C14A—C15A	128.97 (18)	C11B—O5B—C14B—C15B	146.08 (17)
C11A—O5A—C14A—C13A	7.2 (2)	C11B—O5B—C14B—C13B	21.5 (2)
O6A—C13A—C14A—O5A	−102.90 (18)	O6B—C13B—C14B—O5B	−109.88 (18)
C12A—C13A—C14A—O5A	15.1 (2)	C12B—C13B—C14B—O5B	6.4 (2)
O6A—C13A—C14A—C15A	136.89 (18)	O6B—C13B—C14B—C15B	128.32 (19)
C12A—C13A—C14A—C15A	−105.08 (19)	C12B—C13B—C14B—C15B	−115.5 (2)
C16A—O7A—C15A—C14A	−173.67 (18)	C16B—O7B—C15B—C14B	−175.77 (18)
O5A—C14A—C15A—O7A	−66.7 (2)	O5B—C14B—C15B—O7B	−69.6 (2)
C13A—C14A—C15A—O7A	51.9 (2)	C13B—C14B—C15B—O7B	50.2 (2)

### Hydrogen-bond geometry (Å, º)

**Table:**

*D*—H···*A*	*D*—H	H···*A*	*D*···*A*	*D*—H···*A*
N1*A*—H1*A*···N3*B*	0.88	1.92	2.789 (2)	170
O3*A*—H3*A*···O5*A*^i^	0.84	2.07	2.897 (2)	167
O4*A*—H4*A*···O3*B*^ii^	0.84	2.01	2.847 (2)	178
N5*A*—H5*A*···O1*B*	0.88	2.23	3.058 (2)	157
C5*A*—H5*A*1···O1*B*^iii^	0.95	2.63	3.284 (3)	126
C7*A*—H7*A*1···N2*A*	0.99	2.46	3.172 (3)	128
C8*A*—H8*A*···O7*A*^i^	1.00	2.39	3.316 (3)	153
C12*A*—H12*A*···O1*A*^iv^	0.99	2.61	3.432 (3)	141
C12*A*—H12*B*···O1*B*	0.99	2.55	3.426 (3)	147
C16*A*—H16*A*···O4*A*^v^	0.98	2.47	3.401 (3)	158
C16*A*—H16*B*···O6*A*^v^	0.98	2.54	3.222 (3)	127
C16*A*—H16*C*···O2*A*^vi^	0.98	2.50	3.356 (3)	146
C17*A*—H17*A*···O3*A*^vi^	0.98	2.65	3.610 (3)	168
C17*A*—H17*B*···O2*A*^iv^	0.98	2.60	3.573 (3)	175
N1*B*—H1*B*···N3*A*^vi^	0.88	1.94	2.808 (2)	166
O3*B*—H3*B*···O5*B*^vii^	0.84	1.99	2.817 (2)	169
O4*B*—H4*B*···N2*B*	0.84	2.38	3.180 (3)	158
N5*B*—H5*B*···O1*A*^vi^	0.88	2.19	3.027 (2)	159
C5*B*—H5*B*1···O1*A*	0.95	2.60	3.269 (3)	127
C8*B*—H8*B*···O7*B*^vii^	1.00	2.49	3.363 (3)	146
C11*B*—H11*B*···O4*B*	1.00	2.59	3.251 (3)	124
C12*B*—H12*C*···O1*A*^vi^	0.99	2.55	3.363 (3)	140
C12*B*—H12*D*···O1*B*^v^	0.99	2.45	3.424 (3)	167
C14*B*—H14*B*···O4*B*	1.00	2.61	3.272 (3)	123
C17*B*—H17*E*···O2*B*^v^	0.98	2.48	3.456 (3)	176

Symmetry codes: (i) *x*−1, *y*, *z*; (ii) −*x*+1, *y*+1/2, −*z*; (iii) *x*−1, *y*, *z*−1; (iv) *x*, *y*, *z*+1; (v) *x*+1, *y*, *z*; (vi) *x*+1, *y*, *z*+1; (vii) *x*, *y*, *z*−1.
